# A diagnostic tool for malaria based on computer software

**DOI:** 10.1038/srep16656

**Published:** 2015-11-12

**Authors:** Manas Kotepui, Kwuntida Uthaisar, Bhukdee Phunphuech, Nuoil Phiwklam

**Affiliations:** 1 Medical Technology Program, School of Allied Health Sciences and Public Health, Walailak University, Nakhon Si Thammarat, 80161, Thailand; 2 Medical Technology Laboratory, Phop Phra Hospital, Phop Phra District, Tak Province, 63160. Thailand

## Abstract

Nowadays, the gold standard method for malaria diagnosis is a staining of thick and thin blood film examined by expert laboratorists. It requires well-trained laboratorists, which is a time consuming task, and is un-automated protocol. For this study, Maladiag Software was developed to predict malaria infection in suspected malaria patients. The demographic data of patients, examination for malaria parasites, and complete blood count (CBC) profiles were analyzed. Binary logistic regression was used to create the equation for the malaria diagnosis. The diagnostic parameters of the equation were tested on 4,985 samples (703 infected and 4,282 control samples). The equation indicated 81.2% sensitivity and 80.3% specificity for predicting infection of malaria. The positive likelihood and negative likelihood ratio were 4.12 (95% CI = 4.01–4.23) and 0.23 (95% CI = 0.22–0.25), respectively. This parameter also had odds ratios (P value < 0.0001, OR = 17.6, 95% CI = 16.0–19.3). The equation can predict malaria infection after adjust for age, gender, nationality, monocyte (%), platelet count, neutrophil (%), lymphocyte (%), and the RBC count of patients. The diagnostic accuracy was 0.877 (Area under curve, AUC) (95% CI = 0.871–0.883). The system, when used in combination with other clinical and microscopy methods, might improve malaria diagnoses and enhance prompt treatment.

Malaria is a potential medical emergency and should be treated immediately because delays in diagnosis and treatment are leading causes of death in many countries^1^. The first protocol to the diagnosis of malaria infection is a clinical diagnosis based on signs and symptoms of malaria patients which were most widely practiced. The signs and symptoms of malaria include fever, headache, weakness, myalgia, chills, dizziness, abdominal pain, diarrhea, anorexia, and pruritus^2^. However, a clinical diagnosis of malaria from symptoms of malaria patients is still inaccurate because of the non-specific symptoms similar to viral or bacterial infections, and other febrile illnesses which may impair diagnostic specificity^3^,^4^.

To gain a more precise diagnosis, malaria is diagnosed using microscopic diagnosis by the gold standard method which is the staining of thin and thick peripheral blood smears^5^, and other techniques such as the quantitative buffy coat (QBC) method^6^. The rapid diagnostic test (RDT) is another method that detects malaria antigens in a small amount of blood by immunochromatographic assay impregnated on a test strip^7^. The RDT dipstick was a commercially available and frequently uses routine diagnostic tool such as OptiMAL^8^,^9^ ICT^10^, Para-HIT-f^11^, ParaScreen^12^, SD Bioline^13^, Paracheck^14^. Molecular diagnostic methods, such as polymerase chain reaction (PCR) was also used with higher sensitivity when compared to the blood film examination^15^. Some advantages and disadvantages of these methods related to accuracy, precision, sensitivity, specificity, time consumed, cost-effectiveness, and the need for skilled microscopists have been described.

Changes in hematological parameters are well-known features of malaria infection. These changes involve various cell types such as RBCs, leucocytes and thrombocytes^16^,^17^. Malaria infected patients tend to have significantly lower RBC counts, platelets count, leukocyte, lymphocytes count, and Hb level, while monocyte and neutrophil counts were higher in non-malaria infected patients^16^,^17^,^18^,^19^. Nowadays, the gold standard method for malaria detection is examined by expert laboratorists. It requires well-trained labolatorists, it is time consuming, and is an unautomated protocol. In routine laboratory tests, a complete blood count (CBC) is almost always requested without exception as part of the routine investigation in febrile patients. In previously published research, there were some reports using routine automated hematology analyzers for presumptive diagnosis of malaria infection. Most studies are those regarding abnormal depolarizing patterns of the Cell-Dyn hematology analyzer (Abbott Diagnostics, Santa Clara, CA)^20^,^21^,^22^,^23^,^24^. Therefore, this study aimed to develop diagnosis software assisting in the diagnosis of malaria infection in suspected-malaria patients. The software needs to be easy-to-use, include batch screening for routine diagnosis, and have high sensitivity and specificity.

## Results

### Patient characteristics

Between January 1^st^ 2008 and December 1^st^ 2012, data from of 43,899 cases of patients suspected with malaria infection was analyzed. Of these cases, 3,082 (7%) were patients with malaria infection, whereas 40,817 (93%) were patients with non-malaria infection according to thick and thin film examinations.

### Regression model

The regression model was performed to create the equation for malaria diagnosis. In the first, all patients’ parameters previously collected were entered and calculated (Table 1). Variables from the first step that showed significant association with the status of malaria infection (p value < 0.05) such as gender, age, nationality, neutrophil (%), lymphocyte (%), platelet count, monocyte (%), and the RBC count were then entered into the second step of the logistic regression analysis (Table 2). After the second regression analysis, the equation for malaria diagnosis was as shown below:

z = (−7.499) − 0.373(gender) + 0.018(age) + 1.019(nationality) + 0.017(neutrophil) + 0.017(lymphocyte) + 0.022(platelet) − 0.033(monocyte) + 0.691(RBC)

Prob (non-malaria infection) = 1/(1 + (2.178^(−z)^))

Prob (malaria infection) = 1-Prob (non-malaria infection)

Z = constant of regression analysis

**Figure 1:**
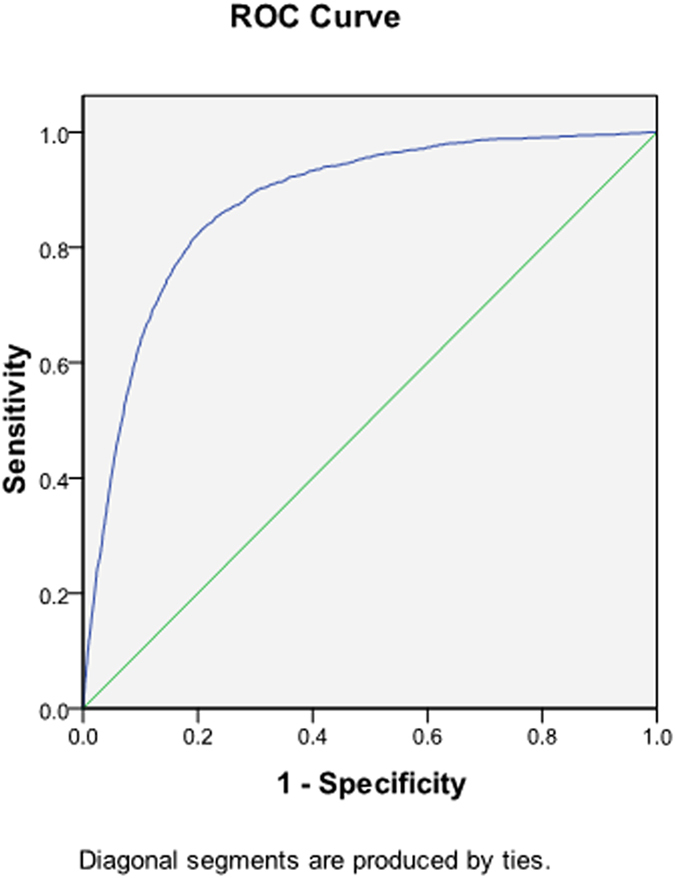
The ROC curve showed the sensitivity and specificity of the equation to detect malaria infection.

After ROC analysis was performed (Fig. 1), the suitable sensitivity and specificity of the equation to detect malaria infections was B value < 0.2208453. The equation had high sensitivity (81.2%) and specificity (80.3%) to detect infection of malaria parasites (Table 3). The positive likelihood ratio was 4.12 (95% CI = 4.01–4.23), while the negative likelihood ratio was 0.23 (95% CI = 0.22–0.25). This parameter also had good odd ratios (P value < 0.0001, OR = 17.6, CI = 16.0–19.3). It indicates that the equation can predict malaria infection after adjust for age, gender, nationality, neutrophil (%), lymphocyte (%), monocyte (%), platelet count, and RBC count of patients. The diagnostic accuracy was 0.877 (area under curve; AUC = 0.877, 95% CI = 0.871–0.883).

## Discussion

In the first regression step, the parameter that was significant in the equation included gender, age, nationality, neutrophil (%), lymphocyte (%), platelet count, monocyte (%), and RBC. This is due to these parameters incurring significant changes during malaria infection that can result differently from non-infected malaria patients. The previous study of patients in Thailand showed that gender, age, nationality, neutrophil (%), lymphocyte (%), platelet count, monocyte (%), and RBC significantly changed from malaria infections. A previous study showed patients with higher a WBC count compared with community controls^10^. The most common complication during malaria infection is thrombocytopenia^6^,^11^,^12^,^13^

An automated blood cell counter (ACC) is a practical tool for malaria diagnosis^21^. In this study, the sensitivity, specificity, likelihood ratio and diagnostic accuracy for all the hematological parameters were determined. The software had high sensitivity and specificity (81.3, 80.1%, respectively) which was higher than the previous study that used a Cell-Dyn® 3500, and analyzed depolarized laser light (DLL) to detect malaria infection, with an overall sensitivity of 72% and specificity of 96%^22^. However, the sensitivity and specificity were lower than the previous study that used a Cell-Dyn® 3500 apparatus to detect malaria pigment (hemozoin) in monocytes, and showed a sensitivity of 95% and specificity of 88%, compared with the gold-standard blood smear^20^. the sensitivity and specificity were lower than the previous study that used a Beckman Coulter ACC to detect increases in activated monocytes by volume, conductivity, and scatter (VCS), with 98% sensitivity and 94% specificity^25^.

Similar to the RDT, this software will reduce the time of malaria diagnosis, with the result obtainable in 5–20 min^7^. In addition, this software can be further used to diagnose malaria in automated instruments. The sensitivity and specificity of this software when compared to RDTs were lower than ParaSight F^26^,^27^,^28^, which yields sensitivity from 77 to 98% and specificity from 83 to 98% for *P. falciparum*. It was also lower when compared to the sensitivity and specificity of the Opti-MAL test^29^ which yields sensitivity of 94% for *P. falciparum* and specificity of 100% for *P. falciparum*. Although the efficiency of this software was lower in terms of sensitivity and specificity than those methods currently used in routine laboratories, it served as another way to help diagnose suspected malaria patients. Further studies are required to improve the sensitivity, specificity, and validate this software. In addition, the software, when used in combination with other clinical and microscopy methods, might help to improve malaria diagnoses in endemic areas.

## Methods

### Ethical approval

This study protocol was approved by The Ethical Clearance Committee on Human Rights Related to Research Involving Human Subjects of Walailak University. The informed consent was not obtained from all subjects but the name and Hospital Number (HN) of patients were not revealed.

### Data collection

The data used in this study were collected from the Medical Technology Laboratory Unit, Phobphra Hospital, Tak Province. The methods were carried out in accordance with the approved guidelines. The medical record keeping system in the Department at The Phobphra Hospital is electronic. The data of all patients diagnosed with malaria between January 1^st^ 2008 and December 1^st^ 2012 were extracted and enter into the SPSS program. Demographic, clinical and laboratory data of all the patients were also collected. Leukocyte, red blood cells, and platelet counts were measured using an automatic cell counter. Other laboratory examinations including RBC, Hb, MCV, MCH, MCHC, RDW, and platelet counts were also collected. The standard procedure used for the diagnosis of malaria is the examination of thick and thin blood smears for malaria parasites by the Wright and Giemsa staining under light microscopy by laboratorists at 1,000 magnification.

### Statistical methods

The Kolmogorov-Smirnov test was performed to test the normal distribution of the data. Binary logistic regression was performed to find out which parameter was suitable for predicting malaria infections; the logistic regression model was performed using the leukocyte count, RBC count, Hb, MCV, MCH, MCHC, RDW, and platelet counts as covariates and using the status of malaria infection (found or not found) as a dependent variable. Data analysis was performed using SPSS ver. 11.5 (SPSS Inc., Chicago, IL, USA). Any parameters that were statistically significant will be put into the second step of regression analysis. Diagnostic accuracy of hematological parameters was measured by a receiver operating characteristic (ROC) and then calculating the sensitivity, specificity, positive/negative predictive values and odds ratios with 95% confidence intervals.

## Additional Information

**How to cite this article**: Kotepui, M. *et al*. A diagnostic tool for malaria based on computer software. *Sci. Rep*. **5**, 16656; doi: 10.1038/srep16656 (2015).

## Figures and Tables

**Table 1:** Logistic regression of demographic and complete blood count variables with the status of malaria infection.

Parameters	B	S.E.	Wald	df	Sig.	Exp(B)
Constant	2.936	0.034	7377.426	1	0.000	18.848
Gender	−0.533	0.088	36.503	1	0.000	0.587
Age	0.013	0.003	26.858	1	0.000	1.013
Nationality	0.814	0.087	86.810	1	0.000	2.256
WBC	0.032	0.014	5.564	1	0.018	1.033
Neutrophil (%)	−0.022	0.006	12.408	1	0.000	0.979
Lymphocyte (%)	0.036	0.006	38.186	1	0.000	1.037
Platelet	0.023	0.001	916.920	1	0.000	1.023
Monocyte (%)	−0.093	0.013	52.435	1	0.000	0.911
Eosinophil (%)	−0.034	0.016	4.692	1	0.030	0.967
Basophil (%)	−0.044	0.055	0.654	1	0.419	0.957
RBC	0.930	0.185	25.381	1	0.000	2.536
Hb	−0.009	0.065	0.017	1	0.895	0.991
MCV	0.020	0.014	1.996	1	0.158	1.020
MCH	0.101	0.045	5.126	1	0.024	1.106
MCHC	0.063	0.028	5.024	1	0.025	1.065
RDW	0.065	0.026	6.043	1	0.014	1.067
Constant	−11.948	1.501	63.387	1	0.000	0.000

**Table 2:** Logistic regression of significant variables with the status of malaria infection.

	B	S.E.	Wald	df	Sig.	Exp(B)
Constant	2.585	0.019	18949.324	1	0.000	13.266
Gender	−0.373	0.048	61.381	1	0.000	0.689
Age	0.018	0.001	174.893	1	0.000	1.018
Nationality	1.019	0.048	457.826	1	0.000	2.770
Neutrophil (%)	0.017	0.004	19.344	1	0.000	1.017
Lymphocyte (%)	0.057	0.004	206.379	1	0.000	1.059
Platelet	0.022	0.000	3502.501	1	0.000	1.023
Monocyte (%)	−0.033	0.007	24.421	1	0.000	0.968
RBC	0.691	0.031	488.879	1	0.000	1.996
Constant	−7.499	0.417	322.765	1	0.000	0.001

**Table 3:** Sensitivity, specificity, PPV, NPV, and diagnostic accuracy of the Maladiag software.

Performance of test	Value
Sensitivity (95% CI)	81.2 (79.8–82.6)
Specificity (95% CI)	80.3 (79.9–80.7)
PPV (95% CI)	23.7 (22.9–24.6)
NPV (95% CI)	98.3 (98.1–98.4)
Positive Likelihood Ratio (95% CI)	4.12 (4.01–4.23)
Negative Likelihood Ratio (95% CI)	0.23 (0.22–0.25)
Diagnostic accuracy (95% CI)	87.7 (87.1–88.3)
